# Theta tACS Over the Medial Frontal Cortex Modulates Executive Function in Parkinson's Disease: A Randomized, Sham‐Controlled Study

**DOI:** 10.1002/cns.71055

**Published:** 2026-07-25

**Authors:** Ke Dong, Yan Zhao, Xiaoxia Zhu, Jiating Li, Bo Peng, Yushu Zhang, Yongxue Li, Guangyan Dai, Dongxu Liu, Yichen Chang, Xi Chen, Hanjun Liu

**Affiliations:** ^1^ Department of Rehabilitation Medicine The First Affiliated Hospital, Sun Yat‐sen University Guangzhou China; ^2^ Guangdong Engineering and Technology Research Centre of Rehabilitation Medicine and Translation Guangzhou China; ^3^ Guangdong Provincial Clinical Research Center for Rehabilitation Medicine Guangzhou China; ^4^ Guangdong Provincial Key Laboratory of Brain Function and Disease Zhongshan School of Medicine, Sun Yat‐sen University Guangzhou China

**Keywords:** executive function, functional near‐infrared spectroscopy, medial frontal cortex, Parkinson's disease, transcranial alternating current stimulation

## Abstract

**Background:**

Executive dysfunction is a common non‐motor symptom of Parkinson's disease (PD) and has been linked to dysfunction of the medial frontal cortex (MFC). This randomized, sham‐controlled study investigated whether high‐definition transcranial alternating current stimulation (HD‐tACS) over the MFC could improve executive function in PD.

**Methods:**

Twenty‐eight PD patients were randomized to receive either active 6‐Hz HD‐tACS or sham stimulation over the MFC for 10 daily sessions. Executive function was assessed using the Digit Span Test (DST), Trail Making Test (TMT), Verbal Fluency Test (VFT), the Simon task, and the stop‐signal task (SST). Functional near‐infrared spectroscopy (fNIRS) was employed to assess prefrontal hemodynamic responses during a separate VFT task.

**Results:**

Relative to sham stimulation, active 6‐Hz HD‐tACS induced significant improvements in TMT‐A, TMT‐B, and semantic VFT performance, along with reduced VFT‐evoked oxyhemoglobin responses in the right dorsolateral prefrontal cortex. Baseline‐adjusted post‐intervention analyses revealed a smaller Simon effect and a higher inhibition rate in the active HD‐tACS group. No significant stimulation‐specific effects were observed for forward or backward DST, phonemic VFT, or SST reaction time.

**Conclusion:**

These findings provide neurobehavioral evidence that 6‐Hz HD‐tACS over the MFC may selectively improve executive function in PD, highlighting the potential of oscillatory neuromodulation for cognitive rehabilitation.

**Trial Registration:**

This trial was registered at ClinicalTrials.gov (No. NCT07240272) on November 20, 2025.

## Graphical abstract

In a randomized sham‐controlled trial, 10 sessions of 6‐Hz HD‐tACS over the medial frontal cortex selectively improved executive function in Parkinson's disease, particularly cognitive flexibility, semantic fluency, and inhibitory control. These behavioral gains were accompanied by reduced right DLPFC activation, supporting oscillatory neuromodulation as a promising cognitive rehabilitation strategy.

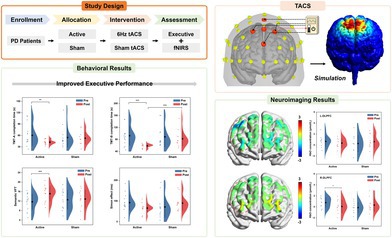

## 1 Introduction

Parkinson's disease (PD) is the second most common neurodegenerative disorder affecting more than 1% of adults aged 65 years and older worldwide [1]. Beyond its cardinal motor features such as bradykinesia, resting tremor, and rigidity [2], PD is often accompanied by cognitive impairment [3, 4], among which executive dysfunction is a prominent feature characterized by deficits in attentional control, working memory, verbal fluency, inhibitory control, and cognitive flexibility [4, 5]. These impairments are associated with reduced daily functioning and poorer quality of life in PD [6]. Nevertheless, the treatment of executive dysfunction in PD remains challenging, as pharmacological interventions yield inconsistent benefits and non‐pharmacological approaches such as cognitive training are constrained by modest effect sizes and methodological heterogeneity [7, 8].

Executive dysfunction in PD has been linked to abnormalities within frontal‐basal ganglia circuits [4, 7, 9]. Within this network, the medial frontal cortex (MFC) is central to performance monitoring, conflict detection, error processing, and adaptive control [10, 11, 12]. Specifically, midfrontal theta activity has been implicated in conflict processing, error monitoring, and adaptive control, with increased MFC theta activity during tasks that place high demands on executive control [11, 13]. MFC theta activity has been thought to coordinate cortical and subcortical regions involved in adaptive control and cognitive flexibility [10]. In line with this view, simultaneous cortical‐subthalamic recordings have shown increased low‐frequency (4–8 Hz) activity in the MFC and subthalamic nucleus (STN) during conflict [14], together with stronger theta phase coherence between the two regions [15]. It is thus plausible that theta activity in the MFC may support executive control through coordination between frontal and subthalamic regions.

However, this midfrontal theta‐related control process appears to be disrupted in PD. For example, reduced midfrontal theta activity in PD has been reported during response conflicts and post‐error processing [16]. In PD, high‐conflict trials are associated with stronger theta phase coherence between the MFC and STN [15], while incongruent trials are associated with increased theta activity in both the MFC and STN [17]. In addition, aberrant theta activity in PD has been associated with deficits in sequential working memory [18] and cognitive impairment including executive dysfunction [19, 20]. Taken together, these findings indicate a close link between abnormal MFC‐related theta dynamics and executive dysfunction in PD.

Transcranial alternating current stimulation (tACS) provides a non‐invasive approach to modulate neural oscillations by applying frequency‐specific external rhythms [21], entraining large‐scale neural networks and inducing lasting plasticity [22]. Considerable evidence suggests that tACS can modulate cognitive performance, including working memory, attention control, executive function, and fluid intelligence [23, 24]. More specifically, theta‐tACS has been shown to modulate executive control in healthy individuals, particularly when applied over medial frontal regions. For example, midfrontal theta‐tACS reduced the Simon conflict effect [25], while 6‐Hz tACS over the dorsolateral prefrontal cortex (DLPFC) reduced the Stroop effect [26]. Midfrontal theta‐tACS has also been found to modulate conflict‐related and post‐error control processes [27, 28]. These findings support the feasibility of targeting the MFC with theta‐tACS to modulate executive control processes.

In this randomized, sham‐controlled study, we examined whether high‐definition tACS (HD‐tACS) over the MFC at 6 Hz could improve executive function in PD. The 6‐Hz stimulation frequency was selected because it falls within the theta range, which has been implicated in frontal cognitive‐control processes and medial frontal–subthalamic dynamics during conflict processing [12, 14, 15]. This selection was further supported by prior tACS studies showing that theta‐range stimulation, including stimulation at 6 Hz, can modulate executive‐control performance [25, 26]. Individuals with PD underwent a comprehensive assessment of executive function before and after the intervention. In addition, functional near‐infrared spectroscopy (fNIRS) was used to assess task‐evoked prefrontal hemodynamic responses. We hypothesized that, compared to sham stimulation, 6‐Hz HD‐tACS over the MFC would lead to improvements in executive function and modulate prefrontal activity in PD.

## 2 Methods

### 2.1 Participants

Twenty‐eight individuals with idiopathic PD were recruited from The First Affiliated Hospital of Sun Yat‐sen University, China (see details in Table 1). All participants were right‐handed native Mandarin speakers and met the United Kingdom Parkinson's Disease Society Brain Bank criteria for idiopathic PD. Eligibility criteria were defined as follows: (1) aged between 35 and 80 years old; (2) absence of other neurological disorders unrelated to PD; (3) no self‐reported hearing impairment; (4) absence of dementia or psychiatric illness; and (5) no history of neurosurgical treatment. Participants were excluded if they were pregnant or had any contraindications to transcranial electrical stimulation. The research protocol was approved by the Institutional Review Board of the First Affiliated Hospital of Sun Yat‐sen University (Approval no. 2021‐885) and conducted in accordance with the Declaration of Helsinki. Written informed consent was obtained from all participants prior to participation. This trial was registered at ClinicalTrials.gov (No. NCT07240272) on November 20, 2025.

**TABLE 1:** Demographic and clinical characteristics of patients. Data are expressed as the mean ± standard error or median (interquartile range).

Characteristic	Active group (*n* = 15)	Sham group (*n* = 13)	Statistics	*p*
Age (years)	64.20 ± 2.39	65.38 ± 2.23	−0.358	0.723
Sex (M/F)	10/5	9/4	0.021	0.885
Disease course (years)	7.76 ± 1.08	7.18 ± 0.85	0.414	0.683
Hoehn‐Yahr stage	2.00 (1.00)	2.00 (0.00)	80.00	0.440
LED (mg/d)	523.10 ± 51.33	528.29 ± 34.08	−0.082	0.936

Abbreviation: LED, levodopa equivalent dose.

### 2.2 Experimental Design

**FIGURE 1:**
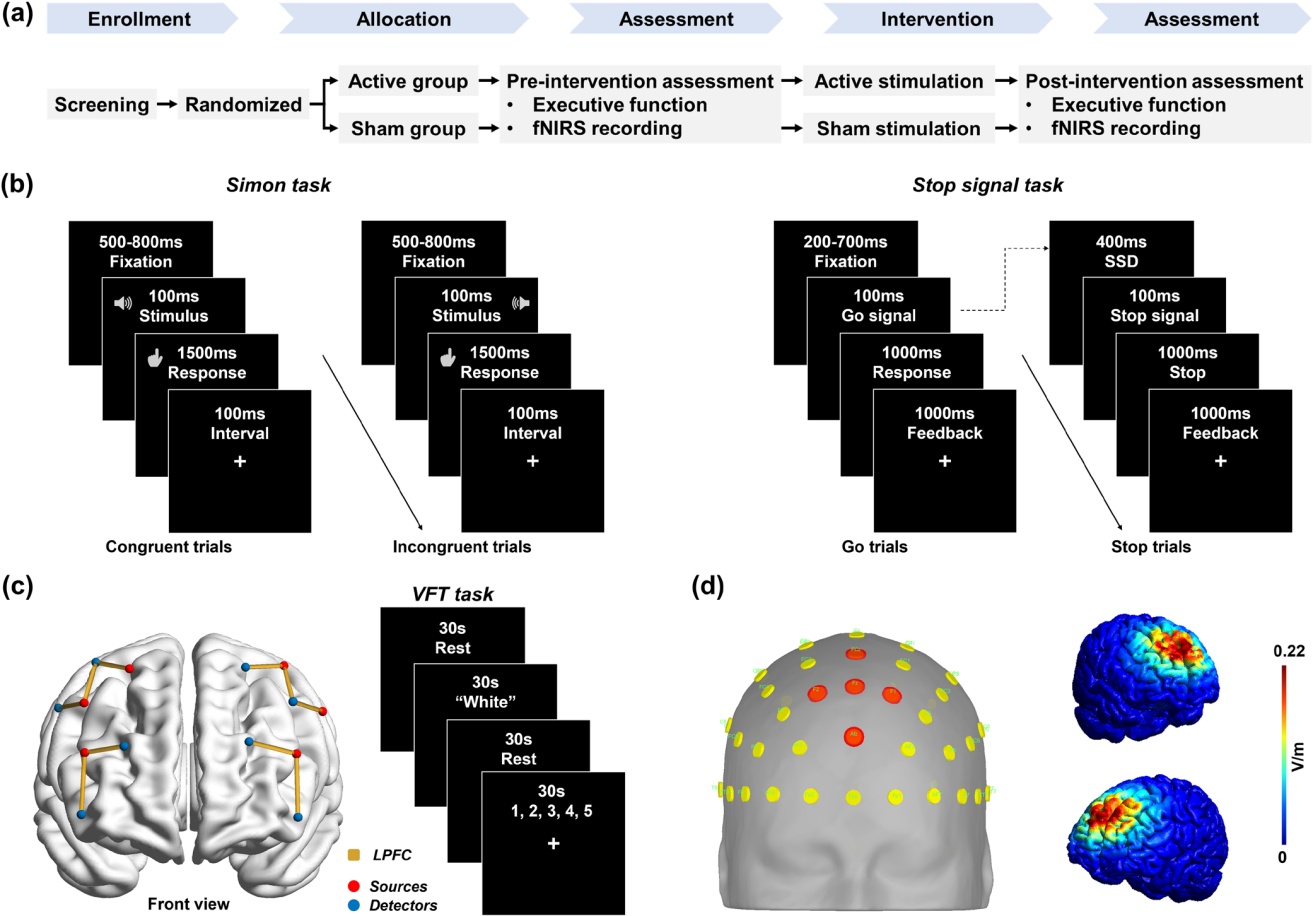
(a) Experimental procedure of the randomized, sham‐controlled study, including enrollment, allocation, pre‐intervention assessment, intervention, and post‐intervention assessment. (b) Inhibitory control paradigms, including the Simon task and stop‐signal task. (c) Block‐designed verbal fluency task during fNIRS recording, with the optode layout in the bilateral LPFC. (d) HD‐tACS electrode montage targeting the medial frontal cortex and the corresponding electric‐field distribution simulated using SimNIBS version 4.6 with the standard head model and the mean stimulation intensity across both groups applied to the central Fz electrode. fNIRS, functional near‐infrared spectroscopy; HD‐tACS, high‐definition transcranial alternating current stimulation; LPFC, lateral prefrontal cortex; SSD, stop‐signal delay; VFT, verbal fluency task.

In this participant‐ and assessor‐blinded, randomized, sham‐controlled study (see Figure 1a), individuals with PD were randomly assigned to either an active stimulation group (age: 64.20 ± 2.39 years; 5 female and 10 male) or a sham stimulation group (age: 65.38 ± 2.23 years; 4 female and 9 male). Randomization was performed using a computer‐generated sequence. Participants and experimenters responsible for outcome assessments were blinded to group allocation. The experimenter administering stimulation was aware of the assigned condition in order to implement the active or sham protocol, but had no role in outcome assessment, data preprocessing, or statistical analysis. As no prior randomized, sham‐controlled studies in this specific line of research were available to support a reliable a priori power calculation for the expected group × session interaction, sample size was determined based on recruitment feasibility and prior comparable neuromodulation studies.

Before intervention, all participants underwent a comprehensive battery of neurocognitive evaluations, followed by fNIRS recordings to assess task‐related cortical activation. Participants then completed an intervention consisting of 10 daily sessions of 20‐min stimulation. They also received standardized motor rehabilitation as part of routine clinical care. Dopaminergic medications were maintained at stable dosages throughout the study, and all assessments were conducted during the medication “ON” state. Post‐intervention assessments were completed after the final stimulation session.

### 2.3 Neuropsychological Assessment

Executive function was evaluated using the Digit Span Test (DST), Trail Making Test (TMT), Simon task, Stop‐Signal Task (SST), and Verbal Fluency Test (VFT). The DST assessed working memory and selective attention using forward and backward digit recall. Participants listened to sequences of digits and repeated them either in the same order (forward span) or in reverse order (backward span). Sequence length increased progressively until failure, and the maximum span correctly recalled was recorded as the score for each condition. The TMT evaluated visual scanning, processing speed, and cognitive flexibility. In Part A (TMT‐A), participants connected 25 Arabic numerals in ascending order as quickly as possible. In Part B (TMT‐B), they alternated between connecting 12 Arabic numerals and 12 corresponding Chinese characters in sequence. Completion time was recorded for each part, with shorter times indicating better performance.

In the Simon task [29], each trial began with a central fixation cross, followed by a 100‐ms pure tone (600 Hz or 1200 Hz) delivered to the left or right ear in a pseudorandomized order (Figure 1b). Participants discriminated stimulus pitch using a two‐button response pad, with response mappings counterbalanced across individuals. Trials were classified as congruent when the response side corresponded to the side of stimulus presentation and incongruent when responses were required on the opposite side. A total of 120 trials were administered, comprising 80% congruent and 20% incongruent trials. The primary outcomes included mean reaction times (RTs) for congruent and incongruent trials, and the Simon effect was defined as the RT difference between incongruent and congruent conditions.

The SST assessed the ability to inhibit an initiated motor response [30]. Each trial began with a fixation cross followed by a 100‐ms “GO” signal (500 Hz tone), prompting participants to generate a rapid motor response (Figure 1b). On 40% of trials, a “STOP” signal (1000 Hz tone) was delivered after a variable stop‐signal delay (SSD), instructing participants to withhold their response. The SSD was initialized at 400 ms and adaptively adjusted between 0 and 900 ms in 50‐ms increments to maintain an approximate 50% stopping success rate, increased after failed inhibition and decreased after successful inhibition. The primary outcomes included the mean RT on GO trials, mean SSD, stop success rate, and stop‐signal reaction time (SSRT). SSRT was calculated by subtracting the mean SSD from the mean GO RT under conditions where stopping accuracy approached 50%.

The VFT evaluated verbal initiation, lexical retrieval, and cognitive flexibility. In the phonemic condition, participants generated as many Chinese words as possible beginning with the syllable “fa” or “xiao” within 60 s. In the semantic condition, participants named as many items as possible from familiar semantic categories (e.g., fruits, vegetables, animals, occupations) within 60 s. Only correct and non‐repetitive responses produced within the time limit were counted in the final score.

### 2.4 fNIRS Recording

fNIRS signals were recorded during the VFT to assess cortical hemodynamic responses associated with the intervention (Figure 1c). Seven of 28 individuals with PD were excluded from fNIRS analysis: three due to intolerance of the fNIRS cap, three due to incomplete post‐stimulation assessment, and one due to motion‐related artifacts. The final fNIRS sample consisted of 21 participants, including 10 participants in the active HD‐tACS group (age: 64.90 ± 6.69 years; 5 female and 5 male) and 11 participants in the sham stimulation group (age: 65.45 ± 8.81 years; 3 female and 8 male). Participants were seated comfortably and instructed to complete the task while minimizing head and body movement. This VFT paradigm, implemented in E‐Prime 3.0 (Psychology Software Tools, USA) during fNIRS recording, differed from the VFT used for behavioral assessment in order to enable reliable estimation of task‐evoked hemodynamic responses. Each block consisted of: (1) a 30‐s baseline rest period, (2) a 30‐s active verbal fluency period in which participants generated words based on visually presented category cues, (3) a 30‐s rest period, (4) a 30‐s control condition involving continuous repetition of numbers 1–5, and (5) a 30‐s rest period. This sequence was repeated three times, resulting in a total duration of approximately 7.5 min.

fNIRS signals were acquired at 11 Hz using a portable dual‐wavelength system (Nirsmart, Danyang Huichuang Medical Equipment Co. Ltd., China), operating at 730 nm and 850 nm. Oxygenated hemoglobin (HbO) signals were analyzed due to their higher signal‐to‐noise ratio and greater sensitivity to task‐evoked cortical activation. Based on the spatial arrangement of the channels and their corresponding cortical projections, 10 channels over the bilateral prefrontal cortex were assigned to two regions of interest (ROIs): the left DLPFC (L‐DLPFC) and right DLPFC (R‐DLPFC). This ROI selection was motivated by the central role of the bilateral DLPFC in the executive control network that supports verbal fluency [31, 32].

### 2.5 tACS Intervention

HD‐tACS was applied over the MFC using a five‐channel stimulator (Soterix Medical Inc., New York, USA). Electrode placement was defined according to the international 10‐10 EEG system, with a central circular Ag/AgCl electrode positioned at Fz, surrounded by four return electrodes at F1, F2, FCz, and AFz (Figure 1d). To visualize the electric‐field distribution generated by this montage, modeling was performed using SimNIBS version 4.6 with the standard head model and a current of 0.38 mA applied to the central Fz electrode, corresponding to the mean individualized stimulation intensity across participants in both groups. Electrodes were secured using a customized cap with plastic holders, and conductive gel was applied to ensure stable contact. Electrode impedance was maintained below 5 kΩ at all sites throughout stimulation. To minimize perceptual or cutaneous sensations, stimulation intensity was individually titrated to the highest level that did not elicit phosphenes or cutaneous discomfort [33, 34]. The individualized stimulation intensity was 0.40 ± 0.16 mA in the active group and 0.36 ± 0.12 mA in the sham group (peak‐to‐peak; mean ± standard deviation), with no significant between‐group difference (*t*(26) = 0.729, *p* = 0.473). During active stimulation, participants received a 6‐Hz sinusoidal alternating current for 20 min with 30‐s ramp‐up and ramp‐down periods. During sham stimulation, participants underwent the same electrode montage, impedance‐control procedure, and 20‐min setup as in the active condition. However, stimulation was delivered only for 30 s at the beginning and 30 s at the end of the session to mimic the transient cutaneous sensations associated with active stimulation [35]. Throughout the intervention period, participants were asked to report any stimulation‐related discomfort or adverse effects. No serious adverse events were observed during the study.

### 2.6 Data Analysis

For neuropsychological assessments, primary outcomes included total correct responses in the VFT, maximum forward and backward digit spans in the DST, and completion times for both TMT‐A and TMT‐B. For inhibitory control tasks, RT data were preprocessed using a median absolute deviation (MAD)‐based outlier detection procedure [36]. In the Simon task, mean RTs were computed for congruent (C) and incongruent (IC) trials, and the Simon effect was defined as their RT difference. In the SST, mean RT for GO trials, mean SSD, and inhibition success rate were calculated, with SSRT estimated as the difference between mean GO RT and mean SSD.

Task‐evoked fNIRS data were processed using the Homer2 toolbox in MATLAB R2022b. Raw signals were converted to optical density and subjected to principal component analysis to attenuate global physiological noise. Motion artifacts were corrected using cubic‐spline interpolation, followed by band‐pass filtering at 0.01–0.1 Hz. Changes in HbO concentrations were then calculated using the modified Beer–Lambert law with differential pathlength factors of [6]. For each participant, HbO signals were segmented into task periods and corresponding rest periods for each block, and task‐related HbO change was computed as the difference between the mean HbO values during the task and rest periods. These values were averaged across blocks to yield channel‐wise estimates and then across channels within each ROI.

### 2.7 Statistical Analysis

Statistical analyses were performed to evaluate the effects of HD‐tACS on executive function using SPSS (v. 20.0). Neuropsychological measures were subjected to two‐way repeated‐measures analyses of variance (RM‐ANOVAs), with group (active HD‐tACS vs. sham stimulation) as a between‐subject factor and session (pre‐ vs. post‐intervention) as a within‐subject factor. Analysis of covariance (ANCOVA) was performed to examine baseline‐adjusted between‐group differences at post‐intervention, with post‐intervention performance entered as the dependent variable, group as the fixed factor, and pre‐intervention performance as a covariate. Greenhouse–Geisser corrections were applied when the assumption of sphericity was violated, and Bonferroni correction was used for post hoc comparisons. All statistical tests were two‐tailed, with a significance threshold of *p* < 0.05. Effect sizes were reported as partial *η* ^2^ or Cohen's *d* values.

For the fNIRS data, HbO activation during the VFT was analyzed at the ROI level (L‐DLPFC and R‐DLPFC). For each ROI, a two‐way mixed ANOVA was conducted with group as a between‐subject factor and session as a within‐subject factor, with *p* values corrected using the false discovery rate (FDR) procedure across ROIs. When a significant interaction between group and session was observed, follow‐up paired‐samples *t*‐tests were performed within each group to characterize session‐related HbO changes, with FDR correction applied.

## 3 Results

### 3.1 Demographic and Clinical Characteristics

Demographic and clinical characteristics of the participants are presented in Table 1. There were no significant between‐group differences in age, sex distribution, disease duration, Hoehn‐Yahr stage, or dopaminergic medication dosage, indicating that active HD‐tACS and sham stimulation groups were comparable with respect to demographic and clinical variables.

### 3.2 Behavioral Performance on the DST, TMT, and VFT

**FIGURE 2:**
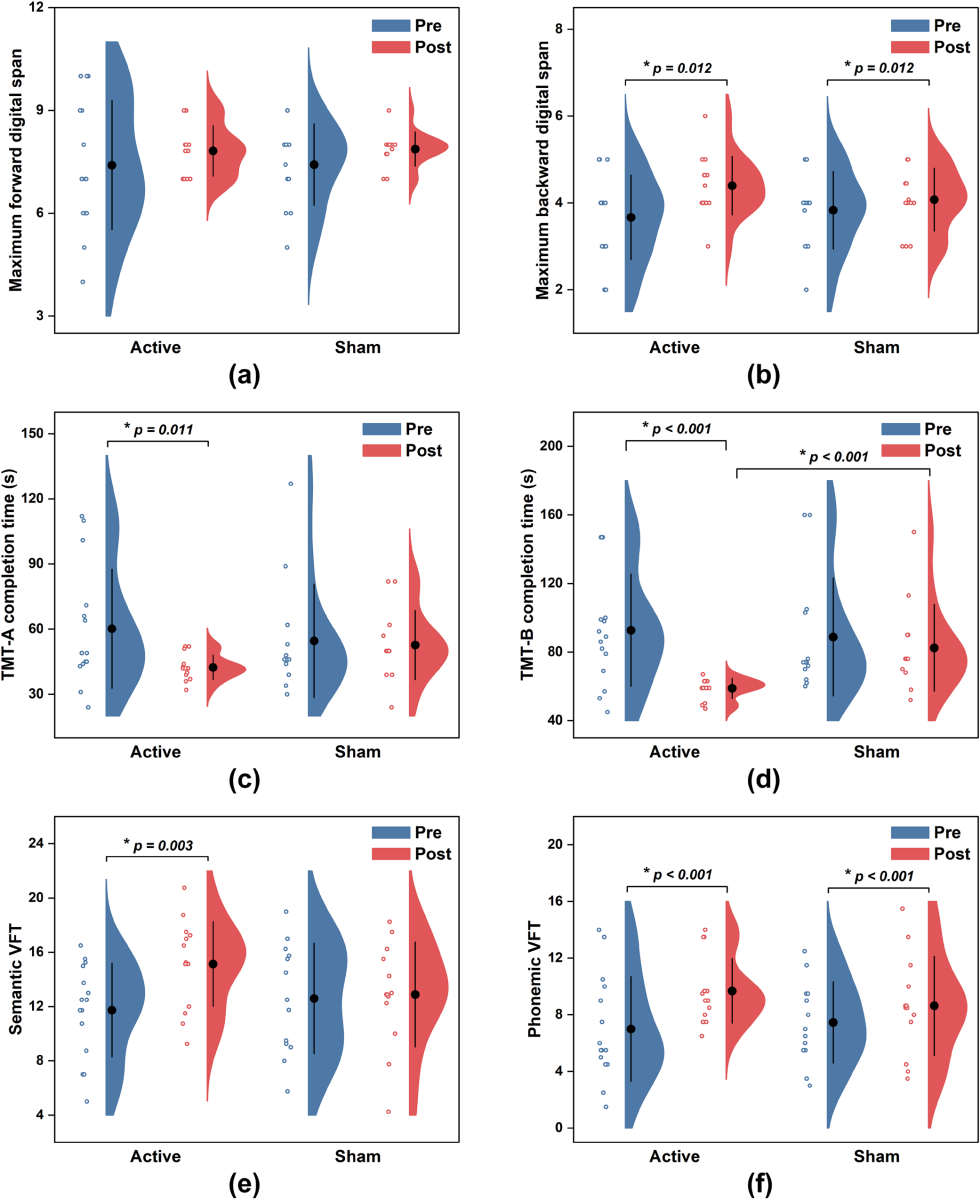
Violin plots show pre‐ and post‐intervention performance in the active HD‐tACS and sham groups for (a) forward digit span, (b) backward digit span, (c) TMT‐A completion time, (d) TMT‐B completion time, (e) semantic verbal fluency, and (f) phonemic verbal fluency. Individual data points are overlaid on each violin, and black dots with error bars indicate the group mean and variability. TMT, Trail Making Test; VFT, Verbal Fluency Test. *indicates a significant post hoc comparison after a significant group × session interaction based on ANOVA.

Figure 2 shows performance on the DST, TMT, and VFT before and after the intervention in the active HD‐tACS and sham stimulation groups. A two‐way RM‐ANOVA conducted on forward DST revealed no significant main effect of session (F(1, 26) = 2.579, *p* = 0.120) or group (F(1, 26) = 0.009, *p* = 0.926) (Figure 2a). No significant group × session interaction was found either (F(1, 26) = 0.004, *p* = 0.948). For backward DST, there was a significant main effect of session (F(1, 26) = 7.306, *p* = 0.012, partial *η* ^2^ = 0.219) (Figure 2b). However, neither the main effect of group (F(1, 26) = 0.091, *p* = 0.765) nor the group × session interaction (F(1, 26) = 1.844, *p* = 0.186) was significant. An ANCOVA conducted with pre‐intervention performance as a covariate revealed no significant main effect of group at post‐intervention (F(1, 25) = 2.090, *p* = 0.161).

A two‐way RM‐ANOVA conducted on the TMT‐A revealed no significant main effect of group (F(1, 26) = 0.191, *p* = 0.666). However, a significant main effect of session was found (F(1, 26) = 4.581, *p* = 0.042, partial *η* ^2^ = 0.150), along with a significant group × session interaction (F(1, 26) = 4.423, *p* = 0.045, partial *η* ^2^ = 0.145) (Figure 2c). Follow‐up analyses showed that the active HD‐tACS group exhibited a significant decrease in completion time following intervention (F(1, 14) = 8.540, *p* = 0.011, partial *η* ^2^ = 0.379). In contrast, no significant main effect of session was found in the sham stimulation group (F(1, 12) = 0.001, *p* = 0.978).

For TMT‐B, a significant main effect of session (F(1, 26) = 16.170, *p* < 0.001, partial *η* ^2^ = 0.383) and a significant group × session interaction (F(1, 26) = 9.296, *p* = 0.005, partial *η* ^2^ = 0.263) were found, whereas the main effect of group did not reach significance (F(1, 26) = 2.124, *p* = 0.157) (Figure 2d). Follow‐up analyses showed that the active HD‐tACS group exhibited a significant reduction in completion time (F(1, 14) = 23.288, *p* < 0.001, partial η^2^ = 0.625), whereas such a modulatory effect did not reach significance in the sham stimulation group (F(1, 12) = 0.539, *p* = 0.477). In addition, the active HD‐tACS group exhibited a significantly shorter completion time than the sham stimulation group at post‐intervention (F(1, 26) = 16.267, *p* < 0.001, partial *η* ^2^ = 0.385), while no such group difference was found at pre‐intervention (F(1, 26) = 0.094, *p* = 0.761).

A two‐way RM‐ANOVA conducted on the semantic VFT showed that, although there was no significant main effect of group (F(1, 26) = 0.307, *p* = 0.584), a significant main effect of session (F(1, 26) = 10.632, *p* = 0.003, partial *η* ^2^ = 0.290) and a significant group × session interaction (F(1, 26) = 7.550, *p* = 0.011, partial *η* ^2^ = 0.225) were found (Figure 2e). Follow‐up analyses showed that the active HD‐tACS group exhibited a significant increase in the number of correct words following intervention (F(1, 14) = 12.338, *p* = 0.003, partial *η* ^2^ = 0.468). In contrast, no significant main effect of session was found in the sham stimulation group (F(1, 12) = 0.374, *p* = 0.552).

For the phonemic VFT, there was a significant main effect of session (F(1, 26) = 13.973, *p* < 0.001, partial *η* ^2^ = 0.350), whereas the main effect of group (F(1, 26) = 0.074, *p* = 0.787) and the group × session interaction (F(1, 26) = 2.130, *p* = 0.156) did not reach significance (Figure 2f). An ANCOVA conducted with pre‐intervention performance as a covariate revealed no significant main effect of group at post‐intervention (F(1, 25) = 2.188, *p* = 0.152).

### 3.3 Cortical Hemodynamic Responses During the VFT

**FIGURE 3:**
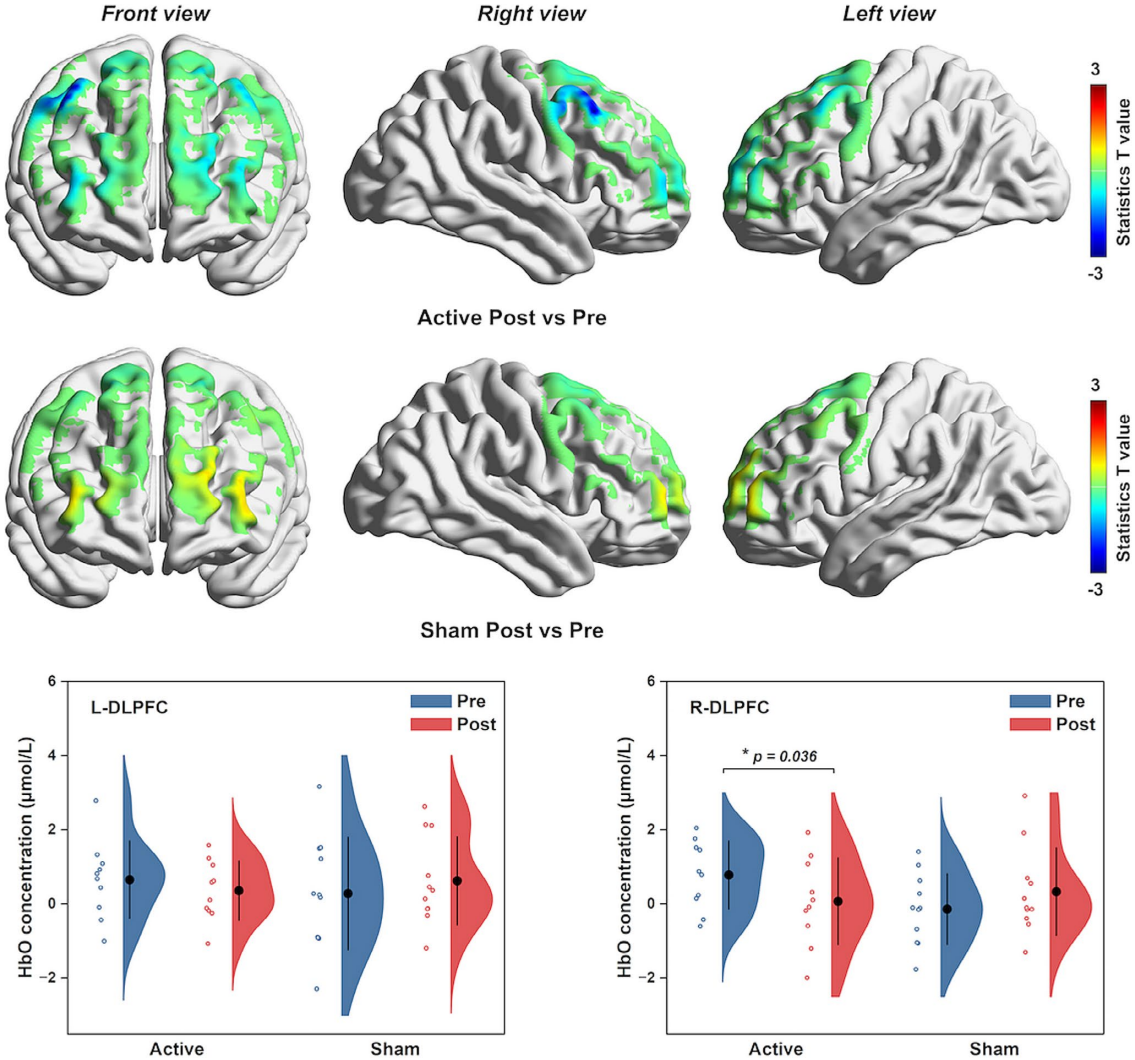
(Upper panel) Cortical surface maps show post‐ versus pre‐intervention changes in task‐evoked HbO responses in the active HD‐tACS and sham groups in the frontal, right lateral, and left lateral views, displayed as statistical *t* values. (Bottom panel) Violin plots show HbO concentrations in the left dorsolateral prefrontal cortex (L‐DLPFC) and right dorsolateral prefrontal cortex (R‐DLPFC) before and after the intervention in the active HD‐tACS and sham groups. A significant reduction in task‐evoked HbO response was observed in the R‐DLPFC after active stimulation, whereas no significant change was found in the L‐DLPFC or in the sham group. Individual data points are overlaid on each violin, and black dots with error bars indicate the group mean and variability. *indicates a significant post hoc comparison after a significant group × session interaction based on ANOVA.

Figure 3 shows post‐pre changes in cortical hemodynamic responses during the VFT as a function of group across the ROIs. In the L‐DLPFC, there was no significant main effect of group (F(1, 19) = 1.26, *p* = 0.276) or session (F(1, 19) = 0.04, *p* = 0.839). The group × session interaction was also not significant (F(1, 19) = 2.89, *p* = 0.106). In the R‐DLPFC, a significant main effect of group (F(1, 19) = 10.73, *p* = 0.008, partial *η* ^2^ = 0.361) and a significant group × session interaction (F(1, 19) = 8.60, *p* = 0.017, partial *η* ^2^ = 0.312) were found, although there was no significant main effect of session (F(1, 19) = 0.22, *p* = 0.839). Follow‐up analyses showed that the active HD‐tACS group exhibited a significant decrease in HbO concentration (*t*(9) = −2.88, *p* = 0.036, Cohen's *d* = −0.91), whereas no significant post‐pre change in HbO concentration was found in the sham stimulation group (*t*(10) = 1.51, *p* = 0.228).

### 3.4 Behavioral Performance in Inhibitory Control

**FIGURE 4:**
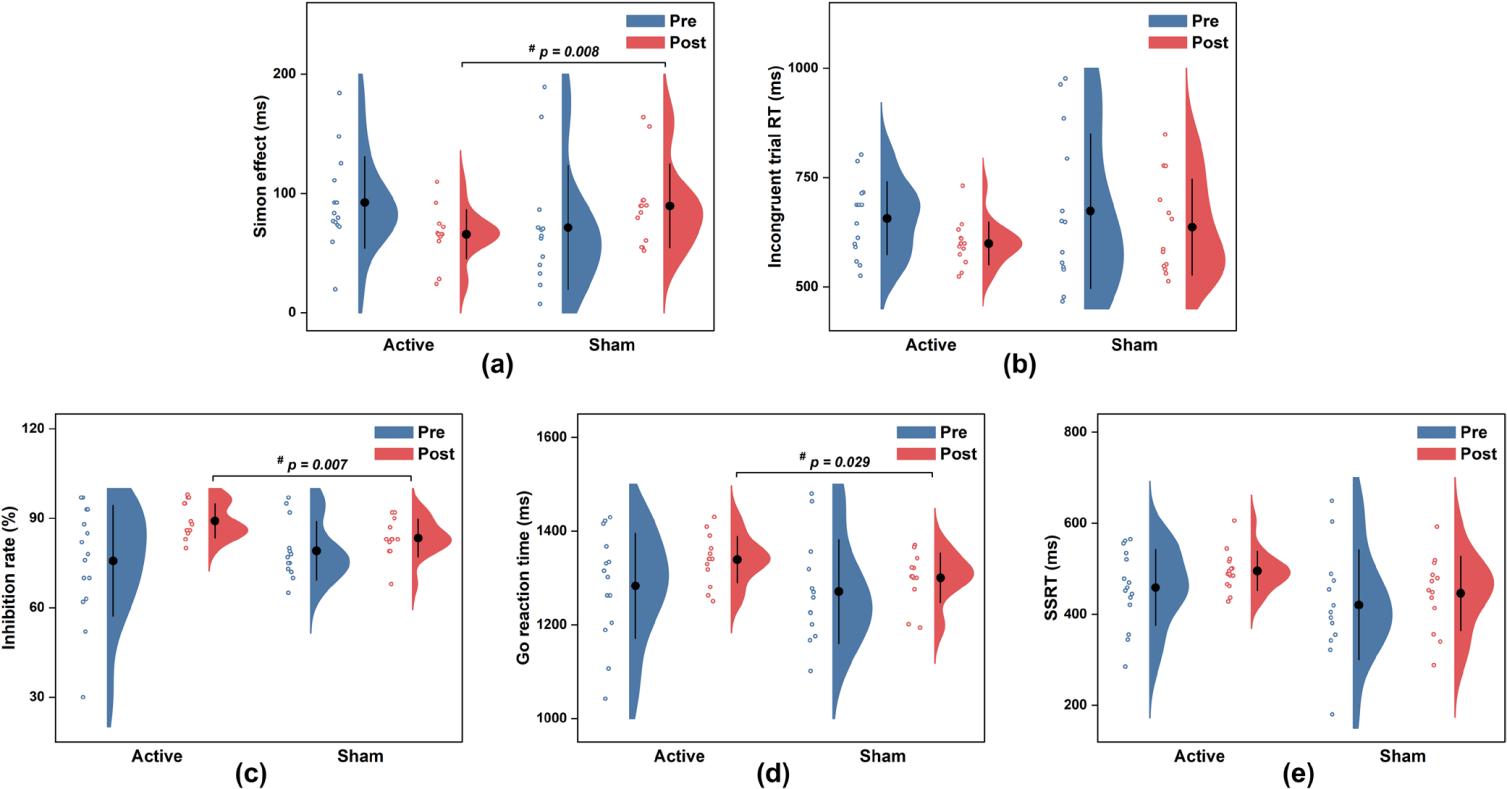
Violin plots show pre‐ and post‐intervention performance in the active HD‐tACS and sham groups for (a) Simon effect, (b) incongruent‐trial reaction time, (c) inhibition rate, (d) Go reaction time (RT), and (e) stop‐signal reaction time (SSRT). Individual data points are overlaid on each violin, and black dots with error bars indicate the group mean and variability. #indicates a significant baseline‐adjusted group difference at post‐intervention obtained from ANCOVA.

Figure 4 shows performance for Simon effect (a), incongruent trial RT (b), inhibition rate (c), Go RT (d), and SSRT (e) before and after the intervention in the active HD‐tACS and sham stimulation groups. For the Simon task, there was no significant main effect of session (F(1, 26) = 2.044, *p* = 0.165) or group (F(1, 26) = 0.478, *p* = 0.496). The group × session interaction (F(1, 26) = 1.442, *p* = 0.241) did not reach significance either. An ANCOVA revealed that the active HD‐tACS group exhibited a significantly smaller congruency cost than the sham stimulation group at post‐intervention (F(1, 25) = 8.341, *p* = 0.008, partial *η* ^2^ = 0.250). For incongruent trial RT, there was a significant main effect of session (F(1, 26) = 8.755, *p* = 0.007, partial *η* ^2^ = 0.252), whereas neither the main effect of group (F(1, 26) = 1.127, *p* = 0.298) nor the group × session interaction (F(1, 26) = 0.207, *p* = 0.653) was significant. An ANCOVA revealed no significant group difference at post‐intervention (F(1, 25) = 0.535, *p* = 0.471).

For inhibition rate, there was a significant main effect of session (F(1, 26) = 11.346, *p* = 0.002, partial *η* ^2^ = 0.304), whereas the main effect of group (F(1, 26) = 0.118, *p* = 0.734) and the group × session interaction (F(1, 26) = 2.991, *p* = 0.096) did not reach significance. An ANCOVA revealed that the active HD‐tACS group exhibited significantly higher inhibition rates at post‐intervention compared to the sham stimulation group (F(1, 25) = 8.649, *p* = 0.007, partial *η* ^2^ = 0.257).

For Go RT, there was a significant main effect of session (F(1, 26) = 6.483, *p* = 0.017, partial *η* ^2^ = 0.200), but the main effect of group (F(1, 26) = 0.821, *p* = 0.373) and the group × session interaction (F(1, 26) = 0.633, *p* = 0.434) did not reach significance. An ANCOVA revealed that the active HD‐tACS group exhibited significantly longer Go RT at post‐intervention compared to the sham stimulation group (F(1, 25) = 5.384, *p* = 0.029, partial *η* ^2^ = 0.177).

For SSRT, there was a significant main effect of session (F(1, 26) = 5.958, *p* = 0.022, partial η^2^ = 0.186), but the main effect of group (F(1, 26) = 2.149, *p* = 0.155) and the group × session interaction (F(1, 26) = 0.179, *p* = 0.675) did not reach significance. An ANCOVA revealed no significant group difference at post‐intervention (F(1, 25) = 3.660, *p* = 0.067).

## 4 Discussion

The present study examined whether 6‐Hz HD‐tACS over the MFC could modulate executive function in PD. Compared to sham stimulation, active HD‐tACS led to improved cognitive flexibility and processing speed, as reflected by shorter TMT‐A and TMT‐B completion times and enhanced semantic VFT performance. In contrast, no significant modulatory effects were found for forward or backward digit span or phonemic VFT. Baseline‐adjusted analyses further revealed a higher inhibition rate and a smaller Simon effect after active HD‐tACS compared to sham stimulation. At the neural level, active HD‐tACS reduced VFT‐evoked HbO concentration in the right DLPFC. These findings provide neurobehavioral evidence suggesting that 6‐Hz HD‐tACS over the MFC may selectively enhance executive function in PD, highlighting the potential of oscillatory neuromodulation for cognitive rehabilitation.

Following 6‐Hz HD‐tACS over the MFC, individuals with PD exhibited shorter TMT‐A and TMT‐B completion times than those receiving sham stimulation. Since prolonged TMT completion has been recognized as a sensitive marker of executive dysfunction in PD [37], this finding suggests improved cognitive flexibility and processing speed after active stimulation. Previous research has linked poorer TMT performance in PD to dysfunction within frontal control systems. For example, reduced activation in the right DLPFC was found during TMT performance in prodromal/early PD [38], while longer TMT completion times were associated with reduced cerebral blood flow in the caudal anterior cingulate cortex [39]. Notably, there is mixed evidence regarding the modulatory effects of tDCS over the frontal region on TMT performance in PD. For example, anodal tDCS over the left or right DLPFC produced sustained improvements in TMT‐B at 1‐month follow‐up [40], and tDCS over the frontal polar area reduced TMT‐A completion time [41]. By contrast, when anodal tDCS over the left DLPFC was delivered concurrently with treadmill‐based aerobic training, improved TMT performance was found in both active and sham stimulation conditions without significant group differences [42]. In the present study, 6‐Hz HD‐tACS over the MFC in PD led to shorter TMT‐A and TMT‐B completion times, extending previous tDCS findings by suggesting that executive performance may be modulated by oscillatory stimulation.

Regarding verbal fluency, 6‐Hz HD‐tACS over the MFC improved semantic VFT performance compared to sham stimulation. However, no such effect was observed for phonemic VFT, although both groups exhibited improved performance after the intervention. Verbal fluency has been widely used to probe executive dysfunction in PD [5, 43]. Semantic fluency relies more on category‐based search and flexible organization of semantic representations, involving interactions between temporal regions and frontal control regions [44, 45]. By contrast, phonemic fluency places greater demands on rule‐guided retrieval under phonological constraints, requiring greater engagement of frontal systems involved in controlled retrieval [44, 46]. Improved semantic VFT following 6‐Hz HD‐tACS over the MFC may therefore reflect modulation of medial frontal control processes, facilitating more efficient selection and switching within semantic categories. This interpretation is consistent with the role of medial frontal theta activity in cognitive control processes, including conflict monitoring, response selection, and inhibitory control [12, 47]. In contrast, the absence of a modulatory effect on phonemic VFT suggests that phonemic retrieval may rely more on phonological search demands and be less sensitive to the medial frontal control mechanisms.

Notably, the active HD‐tACS group exhibited reduced HbO concentration in the R‐DLPFC during the VFT. Verbal fluency engages a distributed frontal‐temporal network, including anterior cingulate and prefrontal regions [44, 45]. In PD, prefrontal activation during the VFT may vary with cognitive status. For example, individuals with PD with preserved cognition showed increased activation across dorsolateral and ventrolateral prefrontal cortices, whereas those with executive‐language impairment exhibited reduced activation in these regions [48]. The reduced R‐DLPFC activation during the VFT following 6‐Hz HD‐tACS over the MFC may be accounted for by the theory of neural efficiency [49], whereby improved behavioral performance accompanied by reduced hemodynamic responses reflects more efficient neural processing [50]. Given the role of medial frontal theta activity in coordinating cognitive control processes [12], it is possible that 6‐Hz HD‐tACS over the MFC may lead to more efficient top‐down control during verbal fluency, facilitating the selection of task‐relevant representations while reducing reliance on the R‐DLPFC. This interpretation is in line with prior evidence showing that improved executive function following intervention is often accompanied by reduced task‐related prefrontal activation [51, 52, 53].

Response inhibition refers to the ability to suppress dominant responses or stop ongoing behaviors [54]. In the present study, the active HD‐tACS group exhibited a higher inhibition rate and longer Go RTs at post‐intervention than the sham stimulation group. However, these effects were identified in baseline‐adjusted post‐intervention comparisons rather than in significant group × session interactions, and therefore should be interpreted as reflecting better post‐intervention performance after active stimulation. This pattern is consistent with a speed‐accuracy trade‐off (SAT) [55], in which successful inhibition is prioritized over response speed. The MFC has been implicated in the regulation of decision thresholds [55]. In PD, low‐frequency activity within fronto‐subthalamic circuits has been linked to increased decision thresholds [56], with higher thresholds associated with slower but more accurate responding [57, 58]. Moreover, applying tDCS over the prefrontal cortex can induce more cautious behavioral choices [59], reflecting a modulation of decision‐making strategy [60]. Therefore, 6‐Hz HD‐tACS over the MFC may increase decision thresholds toward a more conservative response strategy, improving inhibitory success at the expense of response speed.

The Simon task indexes interference from task‐irrelevant stimulus features, and a larger Simon effect reflects greater difficulty in suppressing competing response tendencies [61]. Individuals with PD have been found to exhibit a larger Simon effect with altered delta/theta activity, suggesting disrupted neurophysiological correlates of conflict processing [62]. There is evidence implicating theta‐band activity in conflict processing within frontoparietal control networks. For example, increased task‐related theta activity has been observed under conflict conditions [63], and midfrontal theta activity has been associated with conflict processing and error‐correction dynamics during successful resolution [47]. In the present study, the active HD‐tACS group exhibited a smaller Simon effect at post‐intervention, indicating reduced interference from task‐irrelevant information. Since no group difference was observed for incongruent trial RT, this effect may be related to reduced congruency cost during response selection rather than to a general change in response speed. This pattern is in line with prior evidence in healthy individuals, in which theta‐tACS over the MFC reduced conflict effects in a Simon task on low‐conflict trials [25]. Accordingly, our findings suggest that 6‐Hz HD‐tACS over the MFC may influence conflict‐related control processes in PD.

In addition, 6‐Hz HD‐tACS over the MFC did not improve forward or backward DST performance. In PD, DST impairment can be characterized not only by reduced maximum span length but also by reduced percentage accuracy in conditions that place greater demands on manipulation of stored information [64]. Consistently, conventional span‐based scoring has been shown to be less precise than more fine‐grained digit‐span metrics [65]. It is thus possible that the standard DST measures used in the present study may be insufficiently sensitive to detect small stimulation‐related changes. Alternatively, this null effect may reflect domain specificity of the intervention. Whereas forward and backward DST primarily assess maintenance and manipulation of span‐based working memory [66, 67], 6‐Hz HD‐tACS over the MFC may preferentially influence executive functions more closely related to set shifting, conflict control, and strategic retrieval.

Several limitations should be considered when interpreting these findings. First, the sample size was relatively small, which may have limited statistical power, particularly for outcomes showing only baseline‐adjusted post‐intervention group differences without significant group × session interactions. Second, the fNIRS recordings were obtained using a VFT paradigm that differed from the behavioral assessment, precluding direct brain‐behavior mapping between hemodynamic responses and behavioral performance. Third, in the absence of concurrent electrophysiological measures, it remains unclear whether the observed effects were specifically mediated by modulation of theta‐band activity or by broader changes in the frontal network. Finally, the lack of long‐term follow‐up precludes conclusions about the persistence of the observed improvements. Future studies with larger samples, longer follow‐up periods, and task‐specific neurophysiological measures are needed to further validate the robustness of the observed effects.

## Author Contributions

Ke Dong: conceptualization, writing – original draft, investigation, formal analysis. Yan Zhao: investigation, formal analysis, writing – original draft. Xiaoxia Zhu: writing – original draft, investigation, project administration. Jiating Li: investigation. Bo Peng: investigation. Yushu Zhang: investigation. Yongxue Li: investigation, project administration. Guangyan Dai: investigation, project administration. Dongxu Liu: investigation, project administration. Yichen Chang: investigation, project administration. Xi Chen: writing – review and editing, conceptualization, supervision, project administration. Hanjun Liu: writing – review and editing, conceptualization, supervision, project administration.

## Funding

This study was funded by grants from the National Natural Science Foundation of China (Nos. 82472600, 82172528, 82372556, 82302859, 82402971, 82302848, 82503054), Guangdong Basic and Applied Basic Research Foundation (No. 2023A1515110665), and The Science and Technology Planning Project of Guangdong Province (No. 2023B1212060018).

## Ethics Statement

The research protocol was approved by the Institutional Review Board of the First Affiliated Hospital of Sun Yat‐sen University (Approval no. 2021–885) and conducted in accordance with the Declaration of Helsinki.

## Conflicts of Interest

The authors declare no conflicts of interest.

## Data Availability

The data that support the findings of this study are available from the corresponding author upon reasonable request, subject to institutional agreements and ethical approvals.
